# Stemness, Inflammation and Epithelial–Mesenchymal Transition in Colorectal Carcinoma: The Intricate Network

**DOI:** 10.3390/ijms222312891

**Published:** 2021-11-29

**Authors:** Inese Briede, Dainis Balodis, Janis Gardovskis, Ilze Strumfa

**Affiliations:** 1 Department of Pathology, Riga Stradins University, 16 Dzirciema Street, LV-1007 Riga, Latvia; Inese.Briede@rsu.lv (I.B.); Dainis.Balodis@rsu.lv (D.B.); 2 Department of Surgery, Riga Stradins University, 16 Dzirciema Street, LV-1007 Riga, Latvia; Janis.Gardovskis@rsu.lv

**Keywords:** colorectal cancer, stem cells, inflammation, epithelial–mesenchymal transition, immunohistochemistry

## Abstract

In global cancer statistics, colorectal carcinoma (CRC) ranks third by incidence and second by mortality, causing 10.0% of new cancer cases and 9.4% of oncological deaths worldwide. Despite the development of screening programs and preventive measures, there are still high numbers of advanced cases. Multiple problems compromise the treatment of metastatic colorectal cancer, one of these being cancer stem cells—a minor fraction of pluripotent, self-renewing malignant cells capable of maintaining steady, low proliferation and exhibiting an intriguing arsenal of treatment resistance mechanisms. Currently, there is an increasing body of evidence for intricate associations between inflammation, epithelial–mesenchymal transition and cancer stem cells. In this review, we focus on inflammation and its role in CRC stemness development through epithelial–mesenchymal transition.

## 1. Introduction

**Figure 1:**
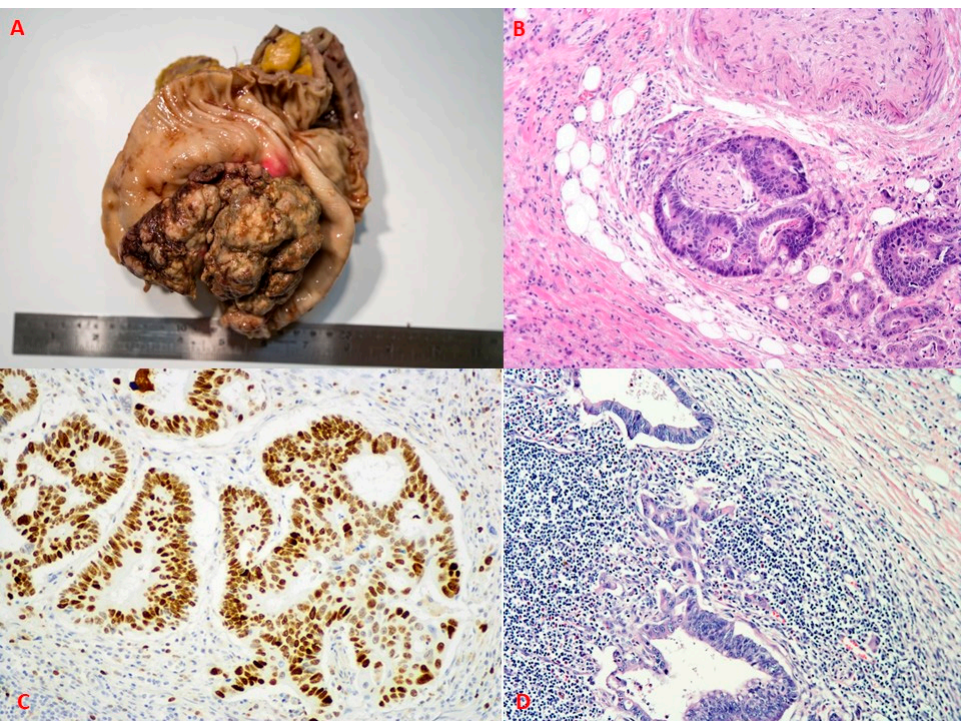
Aggressive features of colorectal carcinoma. (**A**)—Gross view of large, ulcerated colorectal carcinoma. (**B**)—Colorectal adenocarcinoma exhibiting marked perineural growth. Visualisation by haematoxylin and eosin (HE), original magnification (OM) 100×. (**C**)—High proliferation activity by Ki-67. Immunoperoxidase, OM 200×. (**D**)—Metastatic colorectal adenocarcinoma in a lymph node. HE, OM 100×.

Colorectal cancer (Figure 1) has already been targeted by multiple scientific studies, and it remains a hot research topic in oncology. Nevertheless, colorectal carcinoma (CRC) still represents one of deadliest tumours worldwide. According to global cancer statistics (GLOBOCAN estimates), 19.3 million new cancer cases were diagnosed in 2020. CRC represented 10.0% of cancer incidence and caused 9.4% of tumour-induced deaths, ranking third by incidence (after breast cancer, 11.7% and lung cancer, 11.4%) and second among oncological causes of mortality, surpassed only by lung cancer, which was responsible for 18.0% of cancer-induced deaths [1]. Due to the development of diagnostic tools and population screening programs, including colonoscopy, the overall mortality has reduced, in some case-control and prospective cohort studies, by as much as 65–88% [2]. One of the main causes for CRC development is the transformation of a normal colon epithelium into adenomas via multiple genetic and epigenetic aberrations [3]. Removal of adenomas, as a result of a wide screening program, has shown great results in the USA, where the incidence of colorectal carcinoma in people aged 50 years or older declined by 32% (2000–2013) and mortality by 34% (2000–2014) [4]. However, surveillance after adenoma removal is necessary, since CRC incidence in patients with high-risk features of adenomas (Table 1) is at least two times higher than in patients having low and intermediate risk [5]. The current treatment of CRC is mainly based on local surgical intervention, radiation and systemic chemotherapy [6,7,8]. Nevertheless, there are still high numbers of advanced cases, affected by distant metastasis and thus having a worse 5-year survival rate of 10.5% [9]. Multiple problems compromise the treatment of metastatic colorectal cancer, one of these being cancer stem cells (CSCs)—a minor fraction of malignant cells maintaining steady, low proliferation and exhibiting an intriguing arsenal of treatment resistance mechanisms [10,11].

Regarding the origin of CSCs, there are currently two main theories: (1) oncogenic mutations accumulating within normal adult cells or embryonic stem cells, leading to uncontrolled proliferation [12] or (2) dedifferentiation into a stem-like state, which in a cancer cell would produce CSC [13]. Although CSC-targeting treatments are under development, CSCs exhibit heterogeneity that leads to cancer subtype switching, further affecting treatment and prognosis [14].

**Figure 2:**
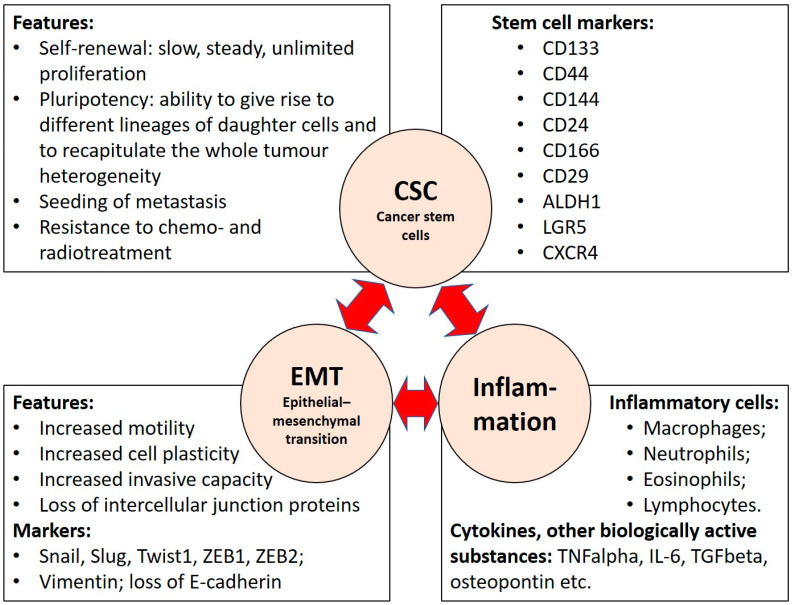
The intricate links between stemness, epithelial-mesenchymal transition and inflammation in cancer.

Inflammation has a well-known role in tumour pathogenesis. Regarding colorectal carcinoma, long-standing inflammatory bowel disease, including both Crohn’s disease and ulcerative colitis, is associated with a 1.4- to 2.2-fold increased risk of colorectal carcinoma [15]. In an already established tumour, the intensity or cellular composition of inflammation can have prognostic importance, and inflammation is also involved in generation of pre-metastatic niches [16,17]. Hence, in colorectal carcinogenesis, inflammation is pathogenetically significant over the whole course of tumour development, from initiation and throughout progression. Relationships between inflammation and cancer stem cells have been demonstrated in different tumours [18,19,20]. In this review we focus on inflammation and its role in CRC stemness development through epithelial–mesenchymal transition (EMT) as shown in Figure 2.

## 2. Stem Cells and Their Markers in Colorectal Carcinoma

The concept of cancer stem cells initially paralleled the hypothesis of normal tissue stem cells. Stem cells are self-renewing cells that maintain a capacity to proliferate, generating new stem cells and daughter cells that undergo differentiation and replenish the pool of functional cells. Stem cells are pluripotent—they can give rise to different lineages of daughter cells. Similarly, the traditional theory of cancer stem cells defines CSCs as a minor fraction of self-renewing malignant cells that maintain a low but steady level of unlimited proliferation. Unlimited proliferation maintains the tumour, but rapid growth is dependent on the fast-dividing progeny of CSCs. The low mitotic activity of CSCs protects them from those treatment modalities that are directed against actively dividing cells. Thus, CSCs can survive treatment and give rise to recurrences [10]. As well as the ability for self-renewal and heterogeneous lineage differentiation, CSCs possess the capacity for clonal tumour initiation, as well as seeding and colonization of distant metastases [11].

Cancer stem cells are also designated tumour-initiating cells (TICs), referring to their capacity to initiate tumour growth and recapitulate the whole heterogeneity of neoplastic tissues, if TICs are transferred to experimental animals. However, TICs are not synonymous with the first malignant cell giving rise to the cancer in human patients.

Although the tumour-initiating ability is important in experimental designs, immunohistochemically detectable stem cell markers have practical value, especially in studies of the formalin-fixed, paraplast-embedded human tissues representing the bulk of surgical pathology samples.

Regarding CRC cell surface antigens, CD133, CD144, CD24, CD44, CD166, CD29, ALDH1, LGR5 and CXCR4 are considered to be CSC markers [21,22,23]. No single marker can identify all CSCs. Data regarding various individual CSC markers are controversial; therefore, some authors have suggested that co-expression of different markers should be evaluated to detect the stemness [24]. However, currently, there is no general set of markers for all CSCs. In addition, studies have shown controversial results regarding tumour progression and the role of CSC markers [24,25].

CD133 is a transmembrane glycoprotein that is involved in cell membrane organization. It has been demonstrated in different types of cancers, where it is closely associated with the Wnt/β-catenin signalling pathway [26,27,28]. In experimental models of CRC, CD133^+^CD44^+^ cells are able to initiate tumours in nude mice [29]. However, some immunohistochemical studies strongly contradict the value of CD133 as a stem cell marker [30,31]. Thus, Czeczko et al. recently reported on a negative (*sic*!) correlation between CD133 expression and death rate, confirmed by univariate analysis. Although the correlation did not reach significance in the multivariate analysis, the trend was opposite to the expected association between stemness and worse prognosis [30]. In addition, Ilie et al. concluded that CD133 alone cannot be used to show CSCs. Several hypotheses can be suggested to explain the difference between in vitro and animal experiments versus studies of human tumours. First, immunohistochemistry is influenced by technological variations regarding: (1) fixation (none versus formalin, pH, time of fixation and cold ischemia); (2) features of the primary antibody (clonality (monoclonal versus polyclonal), clone, concentration, incubation temperature and time); (3) mode (heat-induced versus enzymatic versus none) and features (pH, microwave, temperature, timing) of antigen retrieval; (4) choice of visualisation system; (5) scoring, among others. An indirect hint of technological reasons in the given case is the high proportion of CD133-positive cases observed by immunohistochemistry: 35.9% of all studied cancers and even 44.8% of right-sided carcinomas, as reported by Czeczko et al. 2021 [30]. Further, the inherent complexity of tumour tissues can provide the background for redundant regulatory links. The network of these links could outweigh certain linear biological mechanisms evident in controlled in vitro experiments [32].

**Figure 3:**
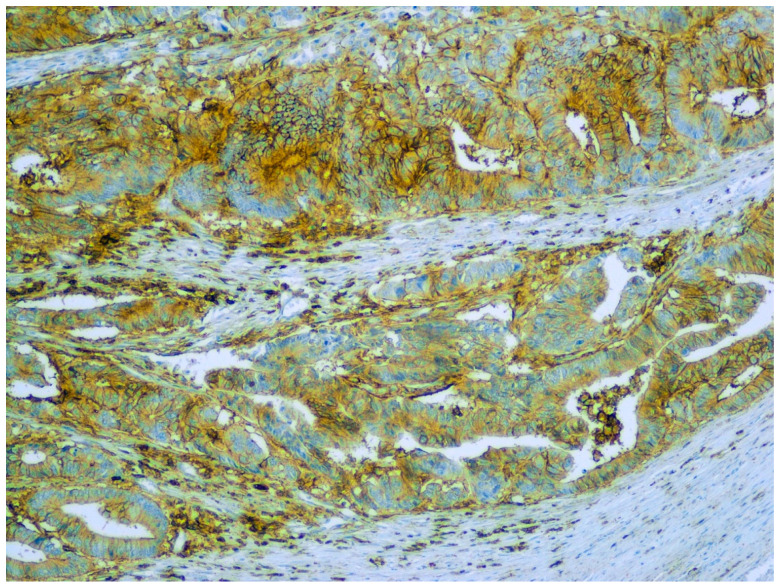
CD44 expression in a colorectal carcinoma. Immunoperoxidase, original magnification 100×.

CD44 (Figure 3) is a transmembrane glycoprotein that normally participates in cell–cell interactions, adhesion of the cytoskeleton to the extracellular matrix and cell migration. In animal experiments, CD44-positive malignant cells show the classic features of TICs. As few as 100 CD44^+^ cells are sufficient to initiate tumour formation in nude mice [33]. In cancers, CD44 is associated with clonality, metastatic spreading ability, resistance to chemotherapy and lower overall survival [24,33,34,35]. Regarding the resistance to treatment, patient-derived organoid analysis has shown an association between the resistance of organoids to 5-fluorouracil and high CD44 expression in carcinomas of the upper gastrointestinal tract [36]. Considering the prognosis, human and animal-based studies have shown CD44 upregulation in bladder cancer, where CD44 overexpression has been associated with the prognostically adverse muscle-invasive basal subtype of bladder cancer [14,37]. Pathogenetically, in several tumour models, up-regulation of CD44 and/or its variants has been linked to inflammation, the relevant mediators and the changes induced by chronic inflammation [22,38].

CD144 is a vascular endothelial cadherin [39]. In cancer studies, it has helped to identify true CSCs. Thus, Yuan et al. found that in mice injected with CD133^+^CD144^+^ TU177 cells, the tumorigenesis rate was significantly (*p* < 0.05) higher than that in mice injected with TU177 cells [40]. Interestingly, in colorectal cancer, CD144 is expressed in endothelial cells generated by CSCs, but also in the tumour itself.

CD24 is a small mucin-like cell surface glycoprotein involved in intracellular signalling [41] and cell clustering [42]. It is overexpressed in malignant cells in comparison with normal tissues. Higher levels of CD24 are associated with worse survival, indirectly suggesting a possible association with stemness [41]. However, several research teams have questioned this correlation [23]. Recently, it has been shown that CD24 overexpression is an early event in colorectal carcinogenesis. Hence, it can represent an oncogene rather than a stem cell marker, although association with stemness is not excluded by these findings [41]. Interestingly, in stem cell clusters, CD24 seems to be expressed in deeper cells located within the cluster. Thus, CD24 may be the adherent molecule connecting the cells within the clusters [42]. Various hypotheses can follow. As Kapeleris et al. suggested, the higher CD24 expression in cell clusters might suggest that the spherical clusters possess more stemness than single cells and therefore are more aggressive [42]. Alternatively, cellular clusters could have evolutionary benefit upon impact of hydrodynamic stress [43] and interaction with platelets and/or inflammatory cells, e.g., neutrophils and macrophages [44]. In this case, CD24 would be functionally important for metastatic spread and thus be associated with worse outcomes even via non-stemness mechanisms.

CD166 is known as an activated leukocyte cell adhesion molecule, belonging to the immunoglobulin superfamily [45]. It is involved in angiogenesis and in haematopoiesis [46]. In the early studies of colorectal carcinoma, membranous expression of CD166 showed a significant correlation with shorter survival [45]. However, the findings of a more recent meta-analysis suggest that CD166 expression decreases the risk of vascular invasion (odds ratio 0.75; *p* = 0.017), and is not associated with overall survival, with the possible exception of stage II colorectal carcinoma [46]. Specific combinations of markers of stem cells and proteins reflecting the activity of β-catenin/mTOR signalling pathways, e.g., CD44/CD166 (*p* = 0.017), CD166/β-catenin (*p* = 0.036), CD44/β-catenin (*p* = 0.001) and CD44/CD166/β-catenin (*p* = 0.001), could be predictors of poor survival in stage II CRC and/or development of liver metastasis [47]. Co-expression of CD29 and CD44 has been tested to enrich the panel of CSC markers, since CD29 is involved in EMT through cross talk with cadherins and CD44 has been reported as marker for CSCs [48].

Aldehyde dehydrogenase 1 (ALDH1) is an intracellular enzyme. As it is involved in the oxidation of cellular aldehydes, ALDH1 activity might influence drug resistance via detoxification processes. In CRC, its immunohistochemical expression has been associated with younger age (*p* = 0.003), gross ulcerations (*p* = 0.01) and the presence of vascular invasion (confirmed by *p* = 0.05) [49].

LGR5 (leucine-rich G-protein coupled receptor 5) is a transmembrane glycoprotein. Upon binding the ligand, LGR5 downstream activates the Wnt/β-catenin molecular pathway leading to increased cellular proliferation [50]. In colorectal cancer, LGR5 has been found in stem cells [51]. Three-dimensional colorectal cancer organoid models, expressing LGR5, showed increased resistance to 5-fluorouracil and irinotecan in comparison with non-stem-cell lines of colorectal cancer [51]. Notably, increased *LGR5* gene expression has already been shown in villous adenomas with a high-grade dysplasia, as well as in an invasive carcinoma, when compared to normal colon cells [52].

CXCR4 is involved in chemotaxis, stemness and drug resistance [53]. Together with LGR5, CXCR4 is considered a marker of CSCs, suggesting that targeting cells containing these markers could improve CRC therapy results [54].

## 3. CRC Stemness and Epithelial–Mesenchymal Transition

Most colorectal tumours originate from epithelium. During carcinogenesis, intercellular contacts are weakened due to loss of intercellular junction proteins, e.g., E-cadherin, β-catenin and others. Malignant cells further acquire mesenchymal features and markers that promote invasion and movement within connective tissues. This cellular plasticity, known as epithelial–mesenchymal transition (EMT), is a key event in distant metastatic spread [21,22,55].

**Figure 4:**
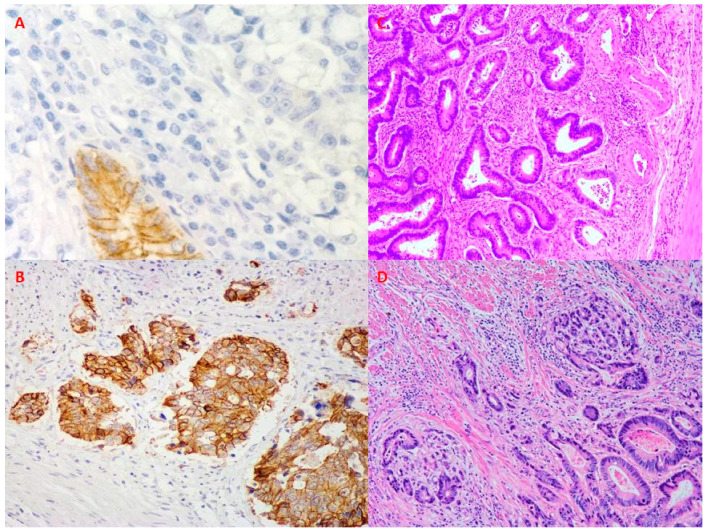
Manifestations of epithelial–mesenchymal transformation in colorectal carcinoma (CRC). (**A**)—Loss of E-cadherin in CRC. Note the retained E-cadherin expression in a normal colonic crypt. Immunoperoxidase (IP), original magnification (OM) 200×. (**B**)—High E-cadherin expression in CRC. IP, OM 100×. (**C**)—Non-budding CRC. Visualisation by haematoxylin and eosin (HE), OM 40×. (**D**)—CRC with florid budding. HE, OM 100×.

In CRC, EMT properties such as cytoskeletal deformability and motility, as well as co-expression of EMT markers, are evident in small cell clusters, known as tumour buds (Figure 4), as reviewed by Grigore et al. [56]. Recently, Sato et al. analysed 32 CRC cases displaying tumour budding. They showed that cancer buds expressed various amounts of LGR5 and PD-L1 and suggested that patients having PD-L1-negative tumour buds should receive different treatment, affecting the CSC marker LGR5 [57]. Thus, tumour budding can be closely associated with stemness. This linkage is not limited to CRC but is likely to reflect a general feature of carcinogenesis. For example, in head and neck cancers, tumour buds have shown EMT properties and increased expression of CSC markers, leading to poor survival rates [58,59]. Tumour budding has been associated with a worse prognosis in various carcinomas [60], paralleling the classic association between stemness and adverse survival.

EMT in a cancer is triggered by complex signalling pathways that include regulation via EMT transcription factors and/or microRNAs (miRNAs) [61]. The relevant transcription factors include Snail, Slug, Twist-related protein 1 (Twist1), Zinc finger E-box binding homeobox 1 (ZEB1) and ZEB2 [62].

Snail family transcription factors are best known in association with EMT. Overexpression of Snail has been associated with down-regulation of E-cadherin, leading to enhanced cell migration and invasion (*p* < 0.002 versus control), as well as significantly higher expression of the CSC markers CD133 and CD44 [63]. Nevertheless, other authors have reported that Snail1 expression in colorectal cancer lacks an association with E-cadherin levels. This could indirectly indicate other ways for EMT induction [64]. Overexpression of transcription factors Snail and Twist1 can also be induced by environmental changes, e.g., hypoxia, where CRC cell lines show changes in the levels of EMT markers such as fibronectin and E-cadherin [65].

The APC/Wnt/β-catenin signalling pathway is widely described in association with EMT and Snail activity [66]. The Wnt signalling pathway is tightly regulated in normal colon stem cells and is one of the most common pathways that is dysregulated in colon cancer, not only in familial cases of CRC but also in the majority of sporadic CRC cases [67]. This pathway participates in a cell cycle in the undifferentiated stem cells in the base of the colonic crypts, allowing survival of both normal and cancer stem cells [68]. Truncation of the APC protein results in enhanced Wnt signalling by β-catenin, which stimulates transcription of Wnt-targeted genes and enhances activation of T-cell factor (TCF) targets, with a subsequent increase in a cell growth, differentiation, spread and adhesion of colorectal cells [69,70]. Disruption in β-catenin function is widely recognized in association with different types of cancer, as well as myofibroblast activation in pulmonary fibrosis [71].

In a mouse model, the link between EMT and CSC has been demonstrated via E-cadherin. Tamura et al. showed that, compared to E-cadherin-negative colorectal CSCs, E-cadherin-positive cancer stem cells have higher tumour growth potential in vivo, via higher expression of the pluripotency factor NANOG, which contributes to elevated levels of cyclins D1 and B1 [72]. In turn, oncogene cyclin D1 is responsible for cell cycle regulation, i.e., progression from the G1 phase of the cell cycle to the S phase [73].

Another mechanism involved in CRC progression is associated with a pro-inflammatory cytokine, TNFα, which is produced by macrophages and CD4-positive T lymphocytes. TNFα has a crucial role in EMT, as it upregulates NF-κB signalling pathways, which leads to overexpression of transcription factors Snail, Slug and Twist, leading to down-regulation of E-cadherin and up-regulation of N-cadherin [74,75]. In addition, it stimulates cell self-renewal by cross action, together with TGF-β [76,77]. TGF-β, produced by activated lymphocytes, induces NF-kappaB activation and EMT, and enhances stemness by CD44 expression in colorectal cancer [78].

Tumour buds are proposed as a morphological sign of EMT in CRC tissues. There is a significant interaction effect for tumour budding between CD44 variant 6 and nuclear β-catenin (*p* = 0.01 by immunohistochemical expression), suggesting that up-regulation of these proteins could contribute to the formation of tumour buds [79]. Yamada et al., in 2017, showed that expression of EMT-related proteins in surrounding stromal cells was significantly higher in areas of high-grade tumour budding than in low-grade areas [80], suggesting that there could be even more complex mechanisms involved within EMT, besides those already known.

The pathways of EMT induction and stemness in CRC differ by microsatellite instability (MSI) status. Twist1 induced EMT and CD44 via AKT/GSK-3β/β-catenin and AKT/NF-kappaB pathways in microsatellite-stable (MSS) cells, while only the β-catenin pathway was activated in MSI colorectal cancer cells [81].

## 4. Inflammation and Its Role in EMT and Cancer Stemness

Within CRC pathogenesis, inflammation plays a significant role, especially in patients with an underlying chronic inflammatory illness such as Crohn’s disease or ulcerative colitis. The risk of CRC-induced death in patients with inflammatory bowel disease is higher than in individuals diagnosed with CRC only, even when adjusting for tumour stage (hazard ratio HR 1.42; 95% confidence interval (CI): 1.16–1.75) [82].

Tumour-related inflammation has been studied in different cancers, targeting also the associations with cancer stemness, particularly in relation to the levels of CD44, known as a cancer stem cell marker. Suwannakul et al. recently described the expression of CD44 within *Opisthorchis viverrini* infection-related cholangiocarcinoma. Higher expression of CD44v9 was found in *Opisthorchis viverrini* infection-related cancers than in cholangiocarcinomas that were not associated with this parasite. There was no CD44v9 staining in the bile duct cells of normal liver. Authors also observed significantly higher expression of inflammation-related markers such as S100P and COX-2 in infection-related cholangiocarcinoma compared to that in non-infected cases and normal liver. These results suggest that stem cell marker CD44v9 may be related to inflammation-associated cancer development [38].

In animal models of breast cancer, CD44 deletion from the malignant cells resulted in delayed carcinogenesis. This finding was accompanied by a decrease in numbers of infiltrating CD206-positive macrophages, known to be associated with tumour-promoting functions [83]. In ovaries, CD44^+^ epithelial ovarian CSCs benefit from the TLR2-MyD88-NFκB pathway, leading to a pro-inflammatory microenvironment that supports stem-cell-driven repair and self-renewal [84].

In a study by Max et al., the relation between tumour budding and inflammation was evaluated in 381 cases of CRC. A combined analysis of tumour budding and inflammatory cell reaction showed that patients with high-grade budding and marked inflammation had a better outcome with respect to both progression-free (*p* < 0.001) and cancer-specific survival (*p* < 0.001) than patients with high-grade budding and only mild inflammation. It was further confirmed by multivariate analysis that in cancers with high-grade budding, marked inflammation is associated with better progression-free (HR 0.59; 95% CI: 0.38–0.92; *p* = 0.021) and cancer-specific (HR 0.58; 95% CI: 0.36–0.93; *p* = 0.024) survival [85]. The association between intense budding and adverse survival has been confirmed by other studies targeting the complex interactions between budding and inflammation/cancer microenvironment in CRC [86]. Stem cell markers, e.g., LGR5, have been shown to be present in the tumour buds [87].

The tumour microenvironment in CRC consists of multiple stromal and immune cell types, including cancer-associated fibroblasts, pericytes, monocytes, macrophages and a subset of T cells [88]. The numbers of CD66b-positive neutrophils are increased in CRC in comparison to adjacent mucosa, and a high density of intratumoural neutrophils is an independent factor for poor prognosis of patients with CRC. High counts of intratumoural neutrophils positively correlated with pT, pM and clinical stage, all with *p* < 0.05 [89].

Although their role in cancer development is still controversial, eosinophils are able to induce cancer cell apoptosis, as shown in direct studies of the interaction between human eosinophils and a colorectal carcinoma cell line. The involved mechanisms include CD11a/CD18 complex, eosinophil cationic protein and eosinophil-derived neurotoxin mechanisms, as well as TNF-α secretion and granzyme A [90]. However, the role of eosinophils in cancer is ambiguous. Thus, for instance, in early studies of cervical cancer, high numbers of eosinophils were found to be associated with worse survival. This finding was explained by a less appropriate immune response, i.e., a disturbed equilibrium between Th1- and Th2-mediated reactions [91,92]. Similarly, marked tissue eosinophilia correlated with an adverse prognosis in Hodgkin’s lymphoma [91,92]. In CRC, increased counts of peritumoural and intratumoural eosinophils are significantly associated with lower T, N and G parameters and absence of vascular invasion, as well as improved progression-free and cancer-specific survival [93]. Prizment et al. showed that high numbers of stromal eosinophils in CRC were associated with lower tumour stage and better overall and cancer-specific 5-year survival, reflected by hazard ratios for death of 0.61 (95% CI: 0.36–1.02; *p* = 0.02) and 0.48 (95% CI: 0.24–0.93; *p* = 0.01), respectively [94].

Tumour-associated macrophages (TAMs) represent an important component of the tumour microenvironment. In a cohort of 81 CRC cases, the relationships of TAMs with EMT markers (E-cadherin and vimentin) were analysed. The TAM antigens CD68 and CD163 were mainly expressed at the tumour invasive front and stroma, while no expression or only weak expression was observed in tumour nests. High levels of CD163 in the invasive front were associated with down-regulation of E-cadherin and up-regulation of vimentin, indicating EMT. Univariate and multivariate analyses indicated that CD163 expression at the invasive front was an independent prognostic factor associated with poor recurrence-free survival (HR 2.414; 95% CI: 1.016–4.523; *p* = 0.045 by multivariate assessment) and overall survival (multivariate HR  =  3.234, 95% CI: 1.176–8.889, *p* = 0.023 by multivariate analysis) [95]. Macrophages are able to produce a wide spectrum of biologically active substances. In vivo and in vitro experiments show that TAMs secrete higher levels of osteopontin when cultivated together with CRC cells. Osteopontin, through binding to its receptor CD44, activates c-jun-NH(2)-kinase signalling and promotes the clonogenicity of CRC cells [96].

There are few studies looking at a stemness and inflammation as a whole. In a study by Xu et al., the correlations between CRC stem cell markers, EMT and immune cells were analysed. Within the tumour centre, the expression of the stemness marker NANOG weakly, but statistically significantly negatively, correlated with CD3- (r = −0.163, *p* = 0.005), CD4- (r = −0.129, *p* = 0.017) and CD8-positive (r = −0.120, *p* = 0.029) T lymphocytes, and expression of LGR5 negatively correlated with CD3-positive T lymphocytes (r = −0.165, *p* = 0.005). There was no correlation between CD68-expressing macrophages and CSC markers in the tumour centre, but high levels of CD44v6 in the invasive front were associated with more CD68-positive macrophages (r = 0.211, *p* < 0.001) [97].

Recently, in a study by Gonzalez et al., the relationships between intratumoural lymphocytes, tumour budding and stromal desmoplastic reaction were described. Intratumoural lymphocytes were associated with low budding (*p* = 0.0247). A competing risk analysis disclosed statistically significant prognostic groups combining intratumoural lymphocytes and budding (*p* < 0.0001). Cases with intratumoural lymphocytes and low budding were associated with better recurrence-free survival compared to cases lacking intratumoural lymphocytes and showing intermediate or high budding (HR 0.214; 95% CI: 0.109–0.421) [98].

In mice models, overexpression of CXCR4 was recently found to promote the EMT and infiltration of myeloid-derived suppressor cells and macrophages in colonic tissue, accelerating colitis-associated and *APC* mutation-driven colorectal tumorigenesis and progression [99]. Other stem cell markers have been shown to be present in a higher amount in CRC budding areas, e.g., a statistically significant relationship was found between the CSC markers Notch1, CD44 and ALDH1 and tumour budding [100].

Oncogenic protein MUC1 is a transmembrane protein with extracellular N-terminal and transmembrane C-terminal subunits. MUC1-C is involved in the Wnt/β-catenin signalling pathway, where it stabilizes β-catenin [101]. MUC1-C induces pro-inflammatory effector TAK1, transforming growth factor β-activated kinase 1. Further, MUC1-C is also linked to activation of the NF-κB pathway, leading to the induction of transcriptional repressor ZEB1, which promotes EMT [102,103]. Finally, MUC1-C activates expression of stem cell marker LGR5 as well as other CSC markers such as BMI1, ALDH1, FOXA1, LIN28B, OCT4, SOX2 and NANOG. These results collectively support the involvement of MUC1-C in driving inflammation, CRC cell stemness and self-renewal capacity [104].

IL-6 is produced by cells of mesenchymal origin. In CRC, CD90^+^ stromal fibroblasts/myofibroblasts produce IL-6, which in turn up-regulates expression of the CSC markers ALDH1 and LGR5 via an IL-6-dependent mechanism. In addition, stromal cells, via IL-6, divert the inflammatory response towards a Th17-driven process favouring tumour growth [105]. However, cancer-associated fibroblasts possess both tumour-promoting and tumour-restraining functions. Selective depletion of α-smooth muscle actin-positive cancer-associated fibroblasts resulted in increased tumour invasiveness, lymph node metastasis and reduced overall survival. The underlying mechanism includes lower production of BMP4 and increased TGFβ1 secretion from stromal cells, which in turn leads to up-regulation of LGR5 CSCs and the development of an immunosuppressive microenvironment with increased frequency of Foxp3^+^ regulatory T cells and suppression of CD8^+^ T cells. Thus, the inflammation and the stemness are regulated via an intricate, balanced biological network of cells and mediators [106].

In studies of cell lines, variations in stemness have been demonstrated in response to chronic inflammation or the removal of it. Chronic inflammation suppressed LGR5 expression, but it was restored after the removal of injurious stimuli. Thus, cancer cells recover their stemness levels after removal of inflammatory stimuli. Chronic inflammation promoted the invasiveness of cancer cells and induced EMT via ZEB, but the invasiveness also decreased to normal levels after the removal of inflammatory stimuli. However, *TP53*-mutated cells had higher cancer stemness and invasiveness than *TP53* wild-type cells under chronic inflammation [107].

The role of inflammation has been studied from the therapeutic point of view. Non-steroidal anti-inflammatory drugs suppress CD133- and CD44-expressing stem cells of colorectal carcinoma via COX-2 inhibition. This effect was evident in a cell culture as well as an in vivo mouse xenograft model [108]. For instance, in breast cancer cell cultures, combined doxorubicin and aspirin treatment significantly reduced the proportion of cancer stem cells and the colony-forming ability. This treatment delayed the inhibition of interleukin-6 secretion, which is mediated by both COX-dependent and COX-independent pathways [109]. Targeting of CD24 might affect several pathogenetic pathways, such as stemness, clustering of circulating cancer cells/CSCS and cytokine environment pathways [110,111]. Interestingly, 470 nm light irradiation inhibited the invasiveness of CD133-positive human colorectal cancer stem cells by suppressing the cyclooxygenase-2/prostaglandin E2 pathway [112].

## 5. Conclusions

Our review shows multiple links between stemness, inflammation and epithelial–mesenchymal transformation in colorectal carcinoma. These interactions are regulated via an intricate, balanced biological network of cells and mediators. The inherent complexity of tumour tissues can provide a background for redundant regulatory links. The network of these links could outweigh certain linear biological mechanisms evident in controlled in vitro experiments. Nevertheless, associations between stemness, inflammation and epithelial–mesenchymal transformation must be recognised when elaborating new treatment regimens, as relatively simple anti-inflammatory treatment can suppress stem cells.

## Figures and Tables

**Table 1:** Risk features for colorectal cancer development after adenoma removal.

High risk 5 or more adenomas <10 mm; 3 or more adenomas with at least 1 ≥ 10 mm; Incomplete colonoscopy/colonoscopy of unknown completeness; High-grade dysplasia; Proximal polyps; Adenomas ≥20 mm;
Intermediate risk 3–4 adenomas <10 mm; 1–2 adenomas with at least 1 ≥ 10 mm;
Low risk 1–2 adenomas <10 mm

## Data Availability

Not applicable.
